# A pictorial review of the utility of CEUS in thoracic biopsies

**DOI:** 10.1186/s13244-020-00944-w

**Published:** 2021-01-28

**Authors:** Gibran T. Yusuf, Cheng Fang, Sa Tran, Deepak Rao, Sam Bartlett-Pestell, Konstantinos Stefanidis, Dean Y. Huang, Paul S. Sidhu

**Affiliations:** 1 Department of Radiology, King’s College Hospital, Denmark Hill, London, SE59RS UK; 2 Department of Respiratory Medicine, Princess Royal University Hospital, Farnborough, Kent, BR68ND UK

**Keywords:** Lung cancer, Contrast-enhanced ultrasound, CEUS, Biopsy, Pleural

## Abstract

Lung cancer is one of the commonest malignancies worldwide and necessitates both early and personalised treatment. A key requirement is histological sampling with immunohistochemistry obtained usually from percutaneous biopsy. Conventionally thoracic biopsies are performed using CT guidance, but more recently, there has been development of physician led ultrasound biopsy for pleural lesions. Contrast-enhanced ultrasound (CEUS) has been increasingly used in interventional procedures and is able to offer benefits for thoracic biopsies including improving lesional visualisation and characterisation, targeting viable tissue and avoiding critical vascular structures as well as evaluating for the presence of post-procedural complications. This educational review aims to benefits of the role of CEUS in thoracic biopsies.

## Key points

- CEUS allows improved lesional characterisation with detailed anatomy.
- CEUS allow accurate mapping for a biopsy path to avoid vasculature.
- Real time CEUS biopsy provides confidence for sampling viable tissue.
- Post procedure complications can be highlighted early through CEUS.

## Introduction

Lung cancer is one of the commonest malignancies worldwide with an estimated 2 million new cases in 2018, accounting for 20% of all cancer deaths [1]. There has been substantial development in both early detection and treatment of lung cancer. In particular, the number of incidentally detected lung nodules has increased significantly with widespread use of computed tomography (CT). Furthermore, with direct access from primary care to chest CT and implementation of lung cancer screening programmes, there will be an increase in lung cancer detection, likely leading to increasing demand for thoracic biopsies.

Managing oncological disease has now become personalised, relying on genetic and immunological profiling necessitating tissue sampling. Commonly tissue is obtained by percutaneous computed tomography guided biopsy (PCTB), which has superseded surgical biopsy in most cases, with decreased complication risk, length of stay and cost of procedure. Although usually a safe procedure, PCTB carries risk of pneumothorax, clinically significant haemorrhage or insufficient sampling [2]. PCTB also requires transfer to the imaging suite, radiation exposure and potentially the administration of iodinated contrast, with further inherent risks. Whilst PCTB can be performed with rapid image acquisition, PCTB is not performed in real time, can be affected by respiration or discomfort causing patient movement and often takes a protracted time in stretched diagnostic services [3].

Ultrasound-guided biopsy (UGB) allows sampling of peripheral located lung and pleural lesions as an alternative to PCTB. UGB offers a real-time method of biopsy with excellent spatial resolution, lack of radiation exposure or use of iodinated contrast, with flexibility of performance in a variety of positions and at bedside. A study comparing UGB and PCTB showed ultrasound to have a decreased procedure time, fewer complications and a likely better pathological specimen, with UGB taking an average 14 min less time than PCTB [3]. Respiratory physicians have adopted pleural biopsy procedures [4] in many centres as the equipment is relatively inexpensive and the procedure can be performed at the bedside, on the ward.

Contrast-enhanced ultrasound (CEUS) has become a well-recognised extension of conventional ultrasound with a myriad of utility [5, 6]. Ultrasound contrast agents (UCA) consist of a phospholipid shell encasing sulphur hexafluoride (an inert gas) measuring approximately the size of a red blood cell. When subject to low acoustic pressure, resonance of the intravenously administered microbubble results in oscillation and nonlinear signal and can be detected in contrast-specific mode. The UCA are truly intravascular agents, and both micro- and macro-vasculature can be seen to provide lesional vascular mapping [5, 6]. UCA are safe in both adults and children with a reaction rate as low as 0.0086% compared to up to 3% with iodinated contrast [7, 8]. It does not cause any nephron or hepatic-toxicity.

CEUS has a well-documented utility in interventional procedures including biopsies [9–11]. At a macrovascular level CEUS allows visualisation of the larger vessels, whilst at a microvascular level, it allows lesional characterisation, increase lesion conspicuity which allows assessment and targeting of areas of active viable disease [9, 10]. For these reasons, utility of CEUS-guided thoracic biopsy is advantageous in comparison with conventional B-mode/colour Doppler ultrasound-targeted thoracic biopsy. This pictorial review will demonstrate the technique, applicability and utility of CEUS-guided thoracic biopsy.

## Technique

CEUS-guided biopsies are still considered a novel technique, and this applies to using this procedure for thoracic lesions. As a consequence, no standardised protocols exist, but the authors describe their preferred approach to ensure safety and success.

### Patient selection

Identification and localisation of a lesion is performed with an initial CT or positron emission tomography (PET-CT), often as part of disease staging. PET-CT is particularly helpful in aiding appropriate selection of the biopsy site, identifying metabolically active sites and components of lesions [12]. Peripheral lung, pleural and chest wall lesions are suitable for CEUS-guided biopsy, however, lesions where intervening aerated lung is present are unsuitable due to the technical limitation of ultrasound; air reflects the ultrasound beam and structures distal are not seen. Conventional contraindications to biopsy remain [2]. The risk of pneumothorax is up to 25% and pulmonary function tests are suggested in order to assess functional reserve should a complication occur [2].

### CEUS Biopsy technique

The patient is positioned appropriately for sonographic evaluation of the lesion, this can often be performed in a sitting or semi-recumbent position. A curvilinear (3–6 MHz) or medium frequency linear transducer (9 MHz) can be used, the latter is of preference to allow better spatial resolution. Conversely, a curvilinear transducer allows better deep visualisation and can help in initial localisation of a lesion. Particularly with the wider field of view obtained.

After optimised B-mode and colour Doppler ultrasound imaging, two bolus injections of UCA are usually required to complete the biopsy procedure. CEUS is first performed for ascertaining the best site for a biopsy to be undertaken. The transducer should be positioned at the approximate tumour midline with an accessible window for a biopsy to be performed, followed by the intravenous administration of 2.4 ml of SonoVue/Lumason™ (Bracco, Milan) via a 21 g cannula ideally placed in antecubital fossa. Imaging of the tumour is undertaken until maximum diagnostic information regarding the tumour vascularity, areas of necrosis and access route are obtained. The bolus of UCA will allow at least 2–3 min to obtain this relevant information. Using a simultaneous imaging of B-mode and CEUS with a spit screen, local anaesthetic is administered and an incision through the skin is performed to allow the biopsy needle access. Then, a second bolus of 2.4 ml of the UCA is administered and in real time, a coaxial 18 g biopsy stylet and needle is advanced into a viable (i.e. vascularised) aspect of the lesion, avoiding any major vessels. The biopsy tract should not traverse aerated lung parenchyma as the needle would be poorly visualised, increasing the risk of procedure. After a sufficient sample is obtained, the needle and then stylet can be removed. An additional bolus of UCA injection can be administered to assess post-biopsy appearances and may identify any active bleeding points; several bolus disease of UCA is safe [7, 8]. If active bleeding is identified, this can be immediately managed by treating the biopsy tract with gelatin sponge or a blood patch. Whilst post-procedure chest radiograph is not always necessary, it is optimally obtained after 2 h prior to discharge for exclusion of pneumothorax.

## Benefits of CEUS-guided biopsy

### Lesion visualisation

**Fig. 1:**
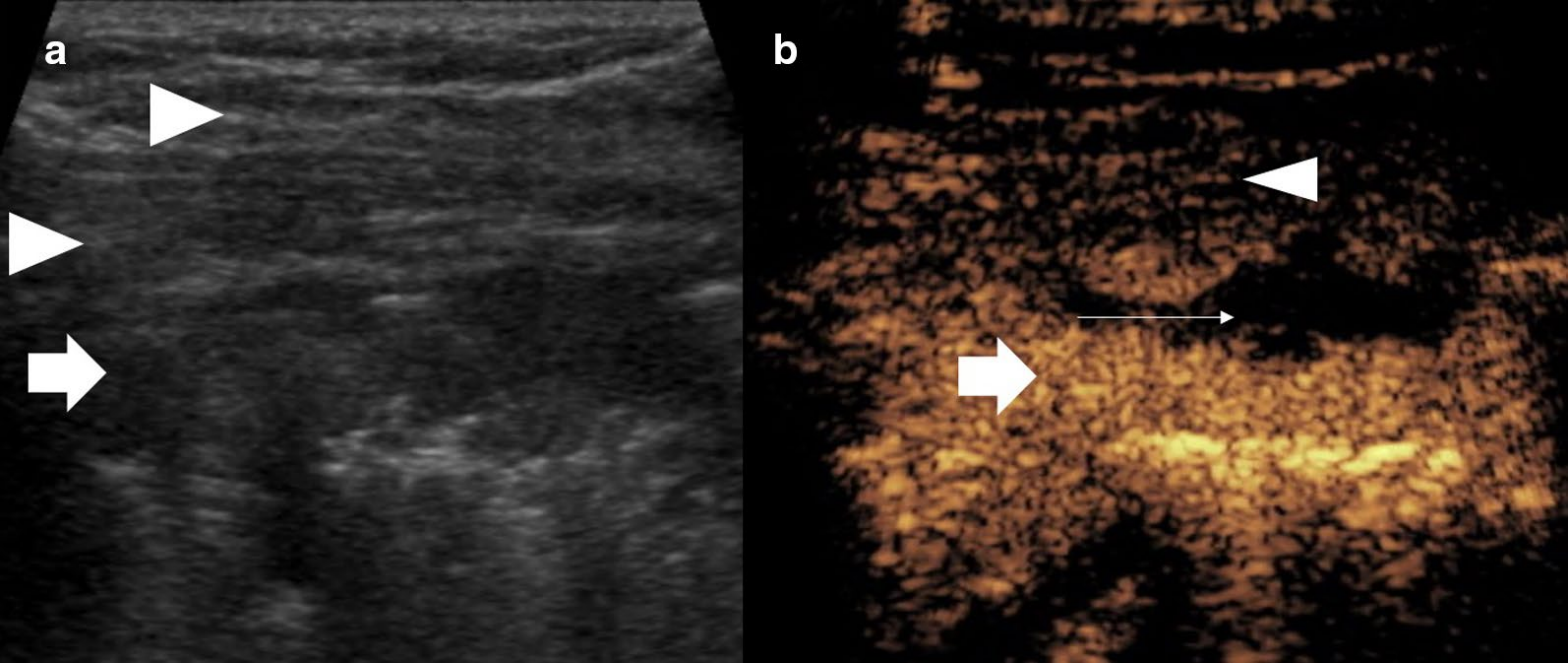
Patient with pleural mesothelioma. **a** B mode ultrasound shows a relatively ill-defined lesion (arrow), which is difficult to differentiate from the striated muscle at some points (arrowheads). **b** CEUS shows clear hyperenhancement of the lesion (arrow) defining the borders and necrotic component (thin arrow) from the straited muscle layer (arrowhead) allowing accurate targeting for an optimal biopsy sample

**Fig. 2:**
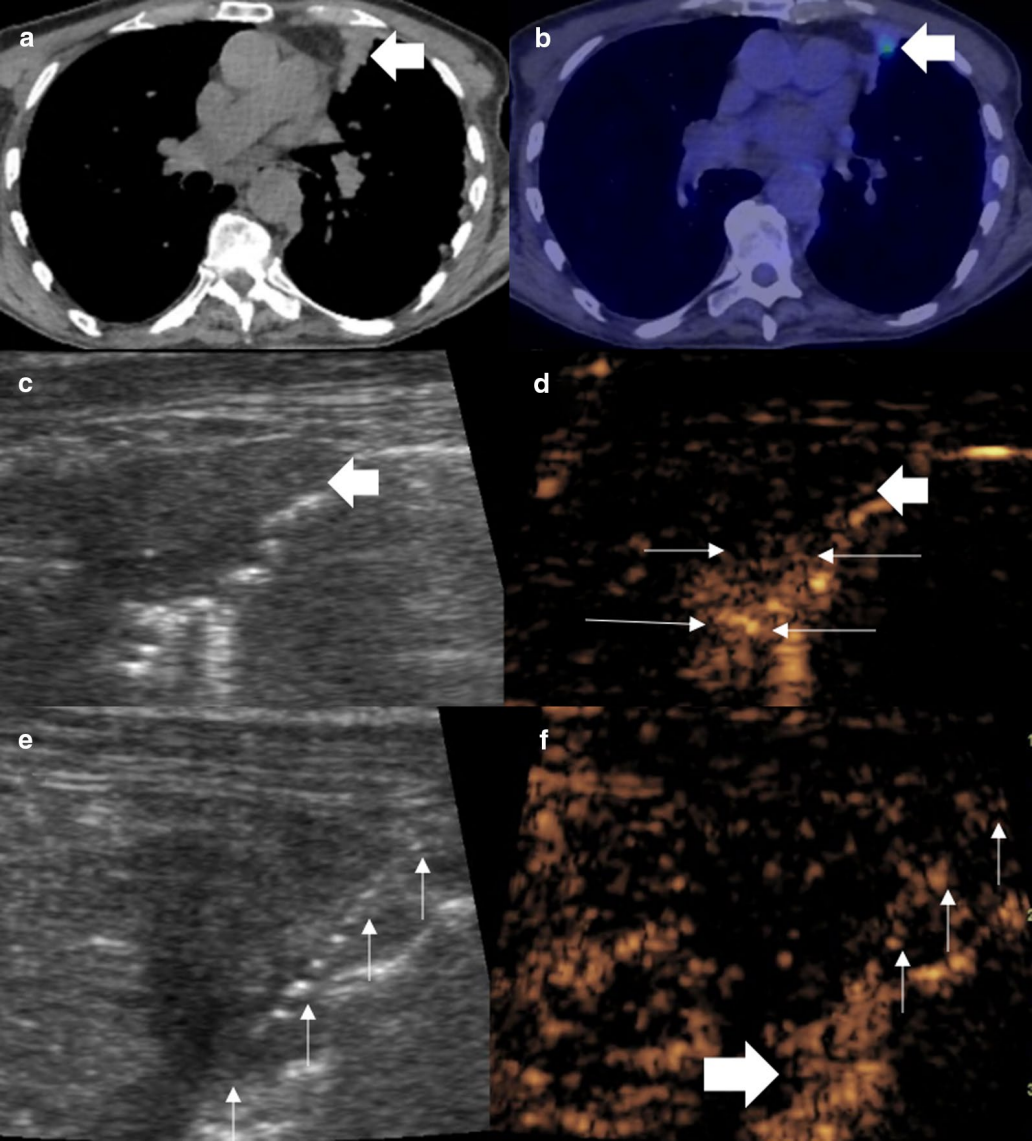
Male patient with persistent cough. **a** A CT examination showed focal anterior pleural thickening (arrow). **b** PET-CT examination demonstrated a small focus of avidity within the pleura thickening. **c** B mode ultrasound defines the area of pleural thickening but not the smaller focus of avidity seen on PET-CT (**d**) simultaneous CEUS showed the subpleural area to be hypoenhancing (thick arrow) with a 4-mm focus of hyperenhancement (thin arrows) suggestive of the pathological tissue. **e**, **f** Simultaneous B mode and CEUS imaging with an 18-gauge biopsy needle (thin arrows) traversed through the enhancing focus (thick arrow). Histology revealed pleural adenocarcinoma

Recognition of a lung or pleural lesion is normally straightforward appearing hypoechoic often with distinct delineation from the adjacent aerated lung or thoracic wall. With an underlying collapsed or consolidated lung, the lesion may be ill defined, with difficulty in assessing the lesion borders. Differentiating thoracic wall lesions may be more problematic (Fig. 1) due to the complexity of striated muscle, and similarly, pericardial lesions or lesions within collapsed or consolidated lung may be inconspicuous (Fig. 2). One of the uses of CEUS is to increase conspicuity of pathological tissue from the background normal tissue, a phenomena dependent on altered vascular perfusion of pathological tissue. The lung parenchyma is predominantly supplied by pulmonary arteries, whereas neovascularity of tumours arises from bronchial arteries [13, 14], with a resulting delayed enhancement of lung tumours comparative to normal lung parenchyma. The neovascularity occurs at 7–20 s post-contrast administration compared to pulmonary arterial supply ≤ 7 s [15, 16]. A further explanation for delayed enhancement is pulmonary hypoxia and vasoconstriction [16]. This different timing of enhancement provides delineation of the pathological process, although there is some overlap between benign and malignant features. Critically, CEUS-guided biopsy has been able to help identify a primary tumour within atelectatic lung [15–17]. The improved conspicuity with CEUS has the added benefit of better delineating lesional borders and identifying cases with chest wall invasion with more confidence (Figs. 1, 2) and avoiding intraparenchymal biopsy. With a good safety profile, repeated UCA administration can allow targeting of the lesion in whichever phase it is most conspicuous. An additional benefit is that previously unseen satellite lesions may become more apparent, which may provide an alternative biopsy path.

### Targeting viable areas

Many tumour types outgrow the neovascularity progression, resulting in areas of necrosis within the tumour, seen in up to 42% of cases [18]. For adequate histological interpretation, tissue with non-viable tumour is often obtained with standard biopsy procedures, results in an insufficient sample particularly as immunohistochemistry analysis requires larger viable specimens. Repeated biopsies carry additional risk as well as delaying treatment.

**Fig. 3:**
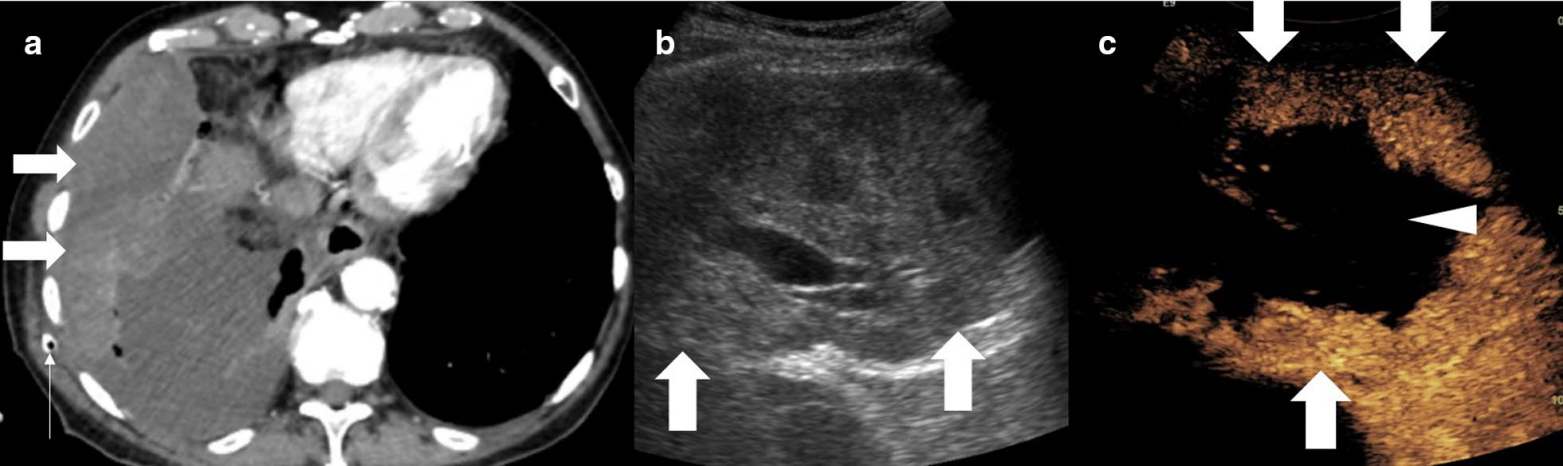
Male patient with recent chest drainage catheter insertion. **a** A CT examination showed focal heterogenous density (arrow) within a pleural effusion, thought to be a haematoma secondary to the drainage catheter insertion (thin arrow). **b** B mode ultrasound of the lesion (arrow) is of a predominantly solid appearance. **c** CEUS defines the large avascular necrotic central component (arrowhead) and a thick enhancing periphery (arrow), indicating that the biopsy had to be taken from the periphery. Histology revealed sarcomatoid mesothelioma

**Fig. 4:**
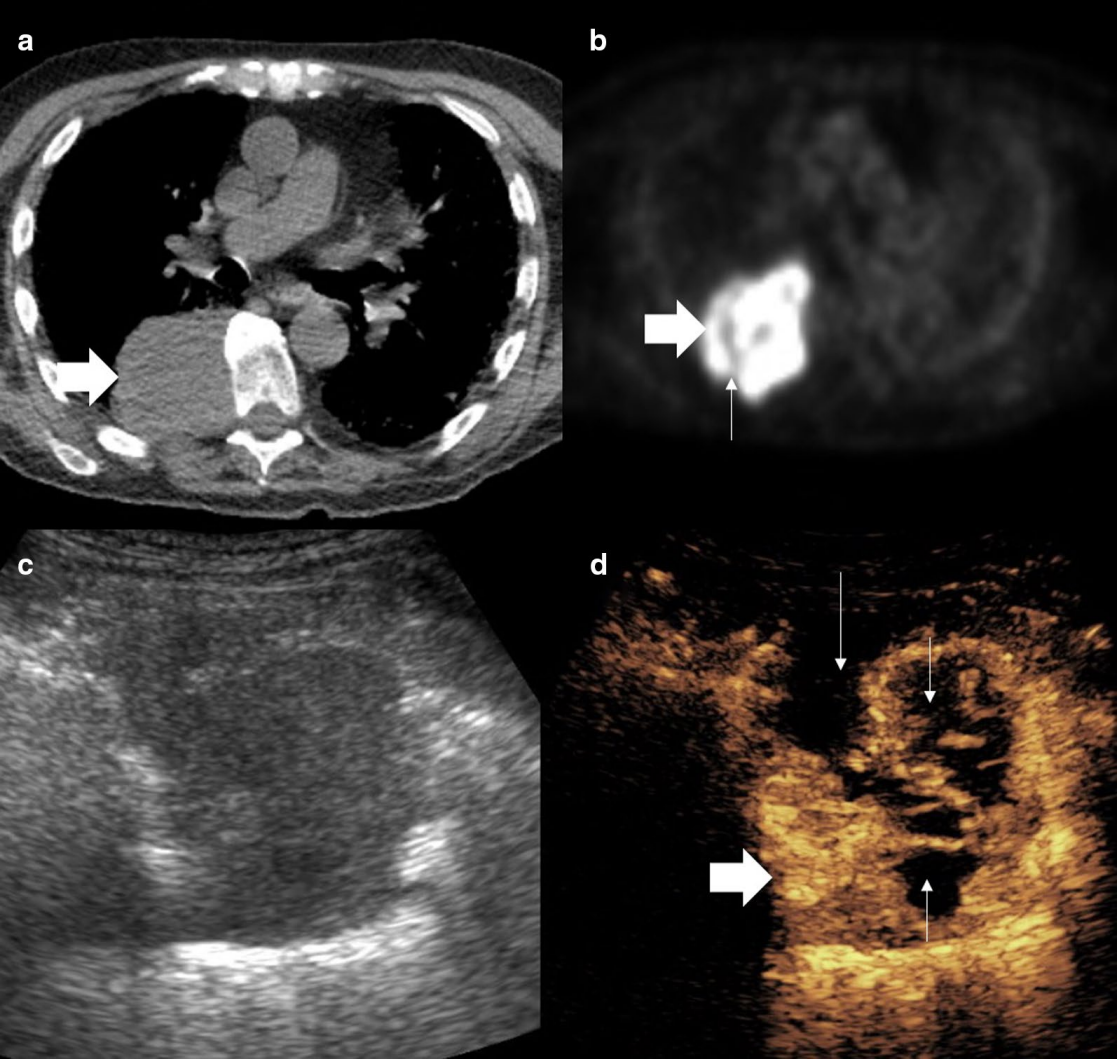
Patient with squamous cell carcinoma. **a** CT examination shows a solid appearing lung lesion (arrow). **b** PET-CT suggests small areas of necrosis (thin arrow) within the avid lesion (arrow). **c**, **d** Simultaneous B mode and CEUS images show the hyperenhancing lesion (arrow) with better appreciation of the complexity and volume of necrosis (thin arrows) indicating a deep eccentric portion required targeting for biopsy (arrow)

**Fig. 5:**
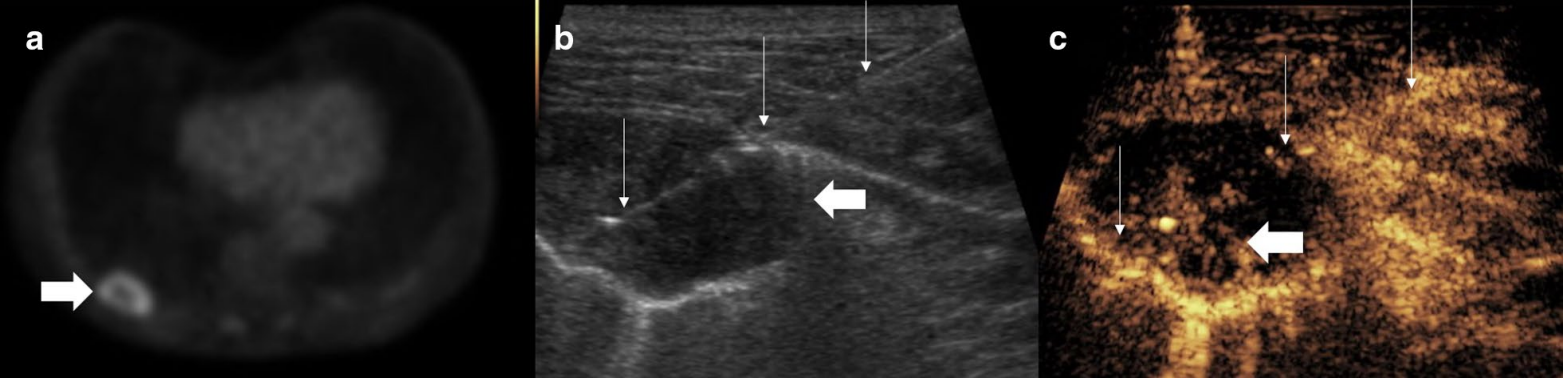
Patient with squamous cell carcinoma. **a** PET-CT shows a peripherally avid lesion with no central avidity (arrow). **b**, **c** Simultaneous B mode and CEUS images as the biopsy is performed. B mode demonstrates a hypoechoic lesion (thick arrow) with the biopsy needle within the lesion (thin arrow). The contrast image (**c**) shows the biopsy (thin arrows) is taken from the deep area of enhancement and therefore viable tissue (thick arrow)

**Fig. 6:**
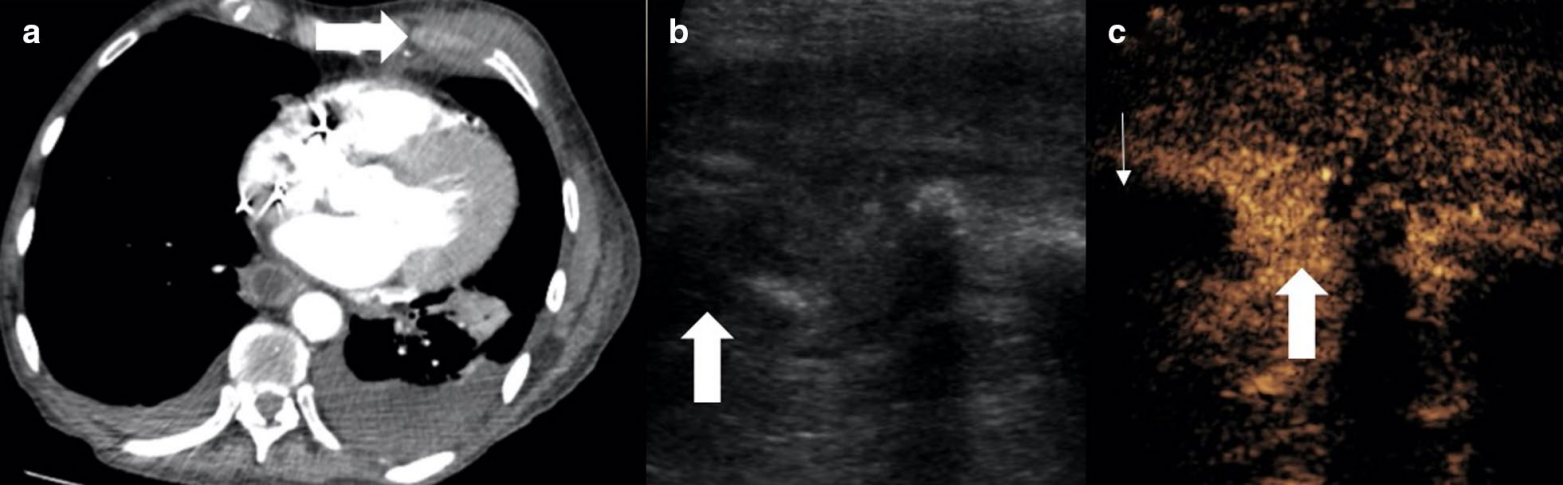
Patient with (**a**) CT examination showing an indeterminate rib lesion (arrow) and clinical sepsis. **b**, **c** Simultaneous B mode and CEUS better defined the lesion which was not well seen on B-mode ultrasound imaging (arrow) into a solid hyperenhancing component (thin arrow) and central avascular component (thick arrow). Fluid aspiration yielded pus of the fluid component and the biopsy was inflammatory indicating osteomyelitis

A CT examination may not have sufficient microvascular detail of lesions in a peripheral location and the snapshot nature of contrast enhancement does not allow for full assessment. As such a PET-CT examination is often required to delineate active disease prior to biopsy [12]. This again does not provide the operator real-time visualisation of the areas of tumour that need to be targeted. The UCA is a pure blood pool agent with transpulmonary stability providing detailed capillary bed assessment. As a result, true vascularity, and particularly the absence of vascularity, can be determined [11, 19, 20]. Conventional ultrasound has been shown to be poor at demonstrating necrotic areas of tumours, with necrosis identified in 7% compared to 44% with CEUS [17]. Importantly, the larger the lesion the greater the degree of necrosis [17]. Whilst a lesion may appear to represent a simple target on CT, the poor contrast differentiation and proportionally greater necrosis may lead to negative sampling. This is particularly true if targeting a peripheral, subpleural aspect of viable tissue within a lesion under CT, owing to the long path potentially required to grant stability [13]. It has been found that the planned needle trajectory was altered in 80% of cases where CEUS was performed in order to avoid necrotic elements [13, 17]. Studies comparing conventional ultrasound to CEUS biopsies have led to significant improvement in diagnostic yield including from 78 to 93% [13, 17]. In addition, safe targeting of viable areas has the potential to further improve diagnostic yield, time taken and number of passes performed—a trend already seen with conventional ultrasound compared to CT biopsy [3]. The improved spatial resolution of CEUS also ensures the correct aspect, containing viable tissue, of even small lesions can be targeted rather than simply targeting the lesion itself (Figs. 3, 4, 5). Conversely, in an infectious lesion, echogenic fluid may be present and borders are ill defined (Fig. 6), also seen in complex effusions. Non-enhancement on a CEUS examination defines fluid elements and can allow fluid aspiration or drainage either for diagnostic purposes of prior to accessing a biopsy site.

### Vascular avoidance

**Fig. 7:**
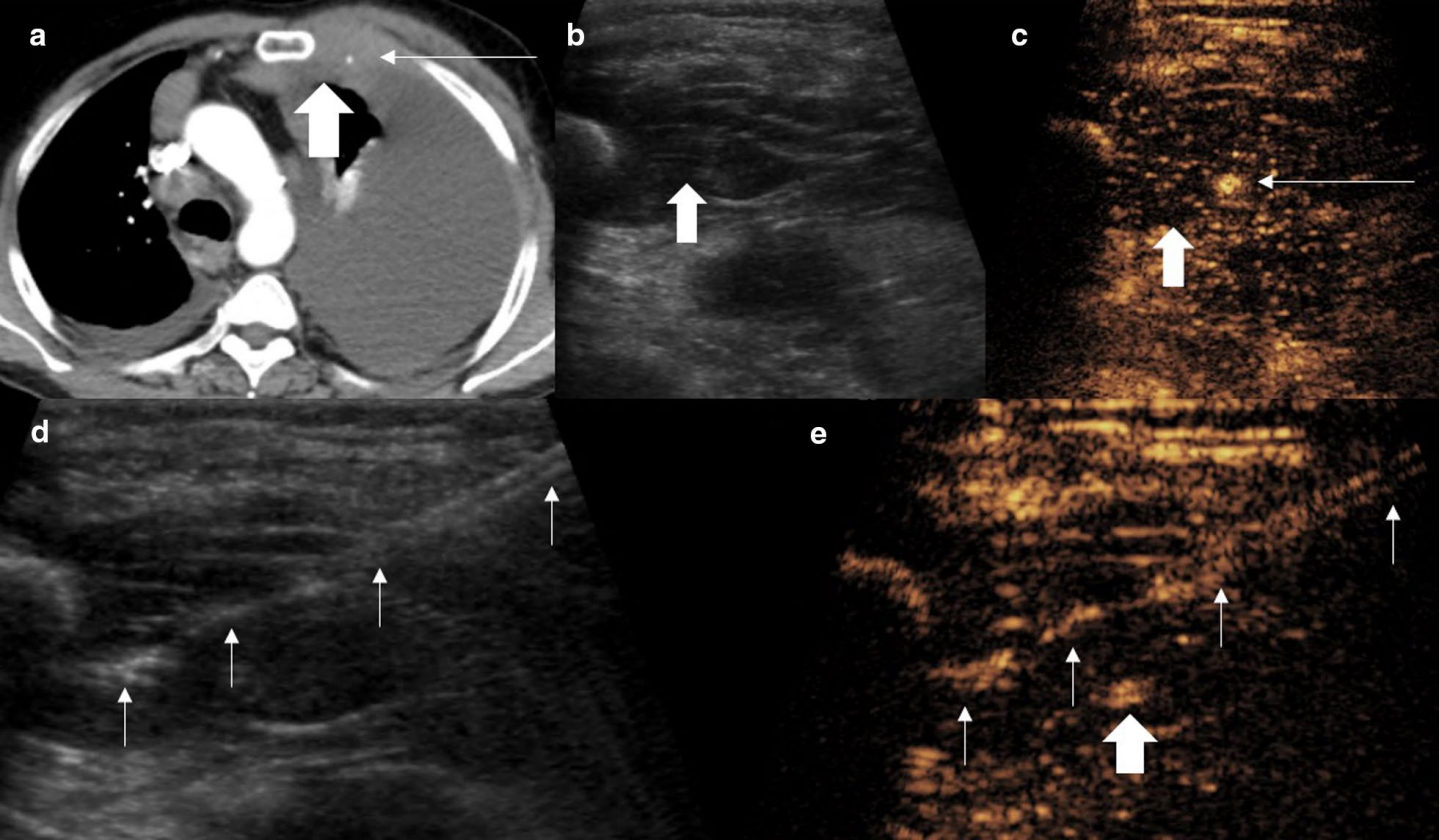
Male patient with chest wall swelling. **a** A CT examination showed focal soft tissue thickening (arrow) encasing the internal mammary artery (IMA) (thin arrow). **b** On the B mode ultrasound, the lesion (arrow) blends with striated muscle but the IMA is not seen. **c** CEUS defines the hypoenhancing pathological tissue (arrow) encasing the IMA (thin arrow). **d**, **e** Simultaneous B mode and CEUS imaging with an 18-gauge biopsy needle (thin arrows) advanced to the lesion avoiding the IMA (arrow). The lesion was histologically proven lymphoma

**Fig. 8:**
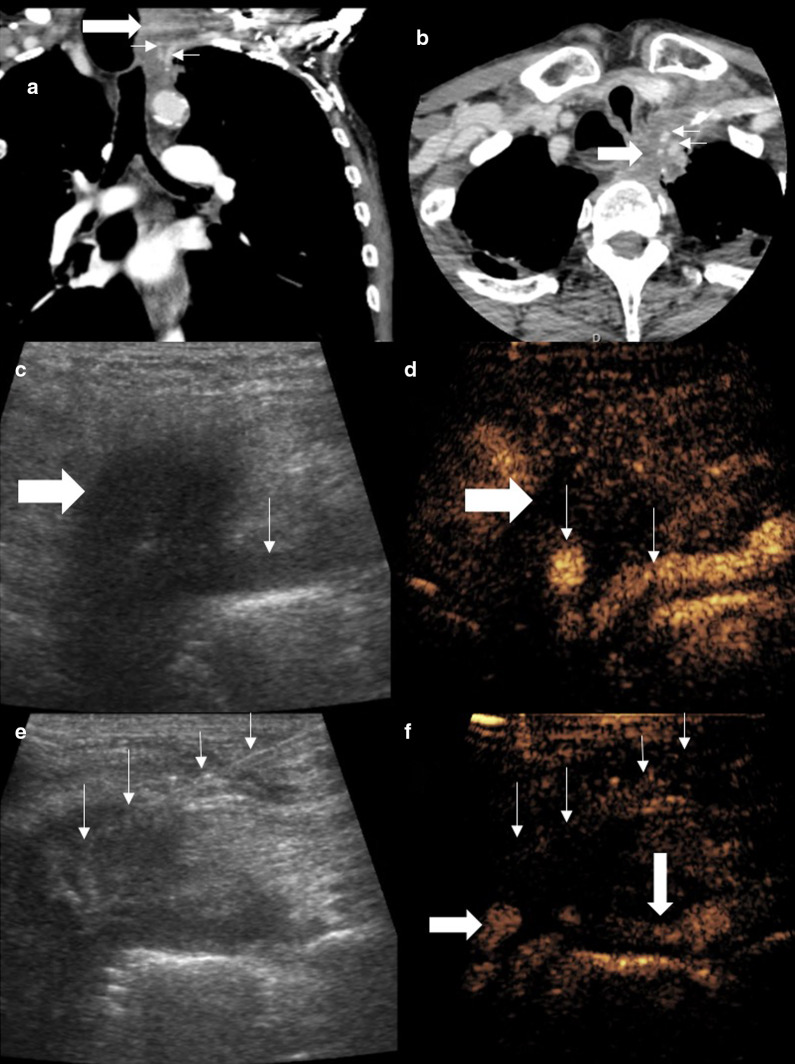
Patient with Pancoast adenocarcinoma of the lung. Coronal (**a**) and axial (**b**) views show soft tissue density (thick arrows) encasing the left carotid artery and subclavian artery (thin arrows). **c** The tumour is seen as a hypoechoic area, ill-defined on B mode ultrasound with the subclavian artery visible on the periphery (thin arrow). **d** Simultaneous CEUS clearly defines the hypoenhancing lesion and the previously unseen carotid artery and subclavian artery (thin arrows). **e**, **f** Simultaneous B mode ultrasound and CEUS with the biopsy needle (thin arrows) within the hypoenhancing lesion avoiding the critical arterial structures (arrow)

Dedicated colour Doppler imaging prior to biopsy often delineates vascular anatomy, most importantly the identification of regional arteries i.e. intercostal or internal mammary arteries. An artery may be difficult to appreciate on B mode ultrasound imaging alone, and the use of colour Doppler during a biopsy creates distracting artefact and is profoundly affected by motion. Colour Doppler ultrasound may be inaccurate in depiction of low flow, particularly if settings are not optimised. Diagnostic CEUS prior to intervention allows identification the micro- and macro-vasculature, and the truly intravascular nature of the UCA ensures recirculation and continued arterial visualisation. At present, no studies have identified a significant difference in bleeding rate post-biopsy with CEUS compared to CT or conventional ultrasound. Typically, intercostal and internal mammary arteries present the most significant risk. Imaging in real time allows avoidance of these vessels (Fig. 7), and it is suggested that CEUS results in improved selection of a needle path [13, 17] (Fig. 8).

### Post-procedural complications

Complications from thoracic biopsy are usually restricted to either haemorrhage or pneumothorax. A pneumothorax is usually readily visible on ultrasound, particularly given the known site of iatrogenic injury. Conventional B mode ultrasound shows the absence of normal pleural sliding and a lung edge may also be seen where a transition point (“lung point”) occurs [22]. However, ultrasound cannot accurately quantify the size of a pneumothorax and conventional modalities of plain radiograph or CT are often required.

**Fig. 9:**
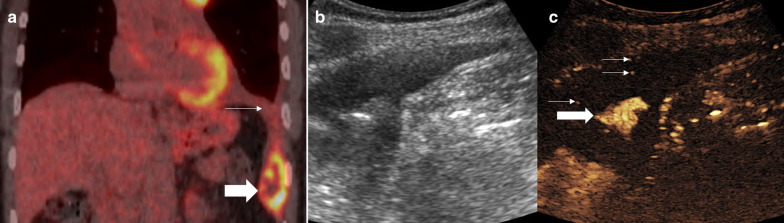
Patient with metastatic squamous cell carcinoma. **a** PET-CT examination shows a peripherally avid lesion in the costophrenic angle (arrow) with an associated pleural effusion (thin arrow). **b**, **c** Simultaneous B mode and CEUS images post-biopsy shows pooling of the UCA within the pleural effusion (thick arrow) which was static on dynamic imaging and single microbubbles (thin arrow) within the otherwise anechoic effusion. The findings are of post-biopsy haemorrhage which ceased without intervention

Haemorrhage may be clearly evident with backflow of blood through a coaxial needle stylet, or may be seen on a post-procedural CT. The post-procedure CT requires a repeat injection of contrast media and may be impractical. CEUS can help in the detection of a focus of haemorrhage, in particular, the presence of a bleeding intercostal or internal mammary artery. The ability of CEUS to define microvacuolar low flow enables accurate depiction of trauma in many organs and has proven utility in identification of active bleeding and pseudoaneurysm formation similar to CT [23]. CEUS features of active haemorrhage are identical to other imaging modalities, shown as an amorphous pooling of the UCA (Fig. 9). In particular, on CEUS, the pooling UCA is clearly seen as a static area and often the bleeding vessel can be identified. If haemodynamic stability is maintained initially following biopsy, a pseudoaneurysm may be appreciated as a contained swirling outpouching of UCA arising from an artery. Interventional treatments can be undertaken, including either direct US-guided thrombin injection or via a trans-arterial catheter angiographic embolisation procedure. Both techniques can utilise CEUS to confirm effective treatment and cessation of bleeding or thrombosis of a pseudoaneurysm [10, 24].

## Conclusion

CEUS presents an additional tool for an ultrasound-guided thoracic biopsy, from a diagnostic and interventional perspective. Histological yield can be maximised whilst making fewer biopsy passes and choosing an optimised path for intervention, avoiding markedly vascularised and obvious necrotic areas. Diagnostic CEUS prior to the procedure can help in characterisation of the lesion, recognising additional features to guide the procedure and reduce complications as well as providing assessment of potential complications post-procedure.

## Data Availability

Not applicable.

## Abbreviations

CEUS: Contrast-enhanced ultrasound
CT: Computed tomography
PCTB: Percutaneous computed tomography-guided biopsy
PET: Positron emission tomography
UCA: Ultrasound contrast agents
UGB: Ultrasound-guided biopsy
